# Impact of Obesity on Sentinel Lymph Node Mapping in Patients with Endometrial Intraepithelial Neoplasia Undergoing Robotic Surgery: A Retrospective Cohort Study

**DOI:** 10.3390/cancers17182972

**Published:** 2025-09-11

**Authors:** Tomer Bar-Noy, Yossi Tzur, Yoav Brezinov, Emad Matanes, Rebecca Lozano-Franco, Shannon Salvador, Melica Nourmoussavi Brodeur, Walter Gotlieb, Susie Lau

**Affiliations:** 1 Division of Gynecologic Oncology, Jewish General Hospital, McGill University, Montreal, QC H3T 1E2, Canada; 2 Department of Obstetrics and Gynecology, McGill University, Montreal, QC H4A 3J1, Canada

**Keywords:** endometrial intraepithelial neoplasia, sentinel lymph node, obesity, robotic hysterectomy

## Abstract

**Background/Objectives**: Lymph node (LN) assessment for cases of endometrial intraepithelial neoplasia (EIN), a known precursor to endometrial cancer (EC), is a topic of debate. Some experts believe this practice could avoid re-staging of disease and influence the decision to administer adjuvant treatment. However, it is known that obtaining sentinel lymph node (SLN) biopsies in patients with an elevated body mass index (BMI) can be more challenging. We thus sought to evaluate the effect of BMI on the SLN detection rate (DR) during robotic hysterectomy in EIN cases. **Methods**: We conducted a retrospective chart review for patients with a pre-operative diagnosis of EIN who underwent robotic hysterectomy with SLN sampling. Five BMI categories were determined according to the literature. Distribution normality was assessed with the Kolmogorov–Smirnov test. Continuous variables, non-parametric continuous variables and categorical variables were assessed with the appropriate statistical tests (two-tailed Student’s *t*-tests, Mann–Whitney U-tests, and chi-squared tests, respectively). **Results**: 115 patients were included (average BMI of 34.75 ± 9.38 SD). The bilateral SLN DR was not significantly different between BMI groups (*p* = 0.606). The difference in unilateral SLN DR between BMI groups was also non-significant (*p* = 0.269). When examining high BMI subgroups (BMI > 30 and BMI > 40), no significant difference was found in bilateral nor unilateral SLN DR. A logistic regression model showed that for every unit of BMI, the likelihood of SLN DR did not change significantly. **Conclusions**: We found no connection between obesity (BMI > 30) or morbid obesity (BMI > 40) and reduced SLN DR in EIN cases, nor a significant variation in the DR when comparing all the different BMI subgroups.

## Simple Summary

The role of lymph node (LN) evaluation in cases of endometrial intraepithelial neoplasia (EIN) remains a topic of debate. Some clinicians choose to perform LN assessment due to the risk of occult endometrial cancer associated with EIN, which has been reported to be as high as 53%. Identifying LN involvement can also provide important information to guide subsequent treatment decisions. This retrospective study aimed to evaluate whether body mass index (BMI) influences LN detection rates during robotic hysterectomy performed for EIN. We analyzed 115 cases from our institution, comparing bilateral and unilateral LN detection across different BMI subgroups. Our findings indicate that BMI did not significantly impact LN detection rates. Detection likelihood remained consistent regardless of BMI category, suggesting that increasing BMI does not impair LN identification during robotic hysterectomy for EIN.

## 1. Introduction

Endometrial intraepithelial neoplasia (EIN), when left untreated, has a 29% chance of progressing to invasive endometrial carcinoma (EC) [1,2,3]. In up to 53% of hysterectomies performed for an EIN diagnosis, the final pathology will reveal concomitant EC [4,5,6]. While lymph node (LN) assessment is included in surgical staging for EC, its performance in EIN is still underexplored. In light of the risk of occult carcinoma at the time of EIN diagnosis (up to 36% with grade 2–3 tumors, and up to 47% with deep myometrial invasion (IB and IC)) [7,8], some surgeons endorse LN assessment at the time of hysterectomy. On the other hand, given the increased morbidity associated with lymph node dissection (LND) [9] and the decreased risk of LN involvement [8], LND performance in EIN is not implemented by other physicians.

Over time, sentinel lymph node (SLN) biopsy has replaced LND as a gold standard technique for LN assessment in EC, offering the advantages of reduced morbidity and preserved staging information [10,11]. Several factors have been associated with reduced detection rate (DR) and detection accuracy of LNs, such as tumor histology and grade, lymph node macro-metastasis, SLN sites, and history of prior pelvic surgery [12,13,14,15]. At our institution, the implementation of SLN assessment in robotic surgery has become the standard of care for patients with EIN since 2010. Matanes et al., in a previous publication, suggested that the SLN might have provided crucial information for appropriately tailoring the therapeutic intervention, and that without this information, treatment decisions might otherwise have been suboptimal [16].

The issue of an increased body-mass index (BMI) and obesity, although widely believed to influence SLN DR, seems to be under discussion due to the differing results of several studies. Although many studies have explored the impact of increased body-mass index (BMI) on the management of EC through minimally invasive surgery, few studies specifically address its effect in robotic surgery for EC. The results of these studies have yielded a variety of debatable outcomes. To the best of our knowledge, no similar study on the effect of BMI on SLN detection in the context of robotic hysterectomy for EIN has been published. In this study, we sought to evaluate the correlation and effect of BMI on SLN DR during robotic hysterectomy in the presence of EIN.

## 2. Materials and Methods

**Figure A1:**
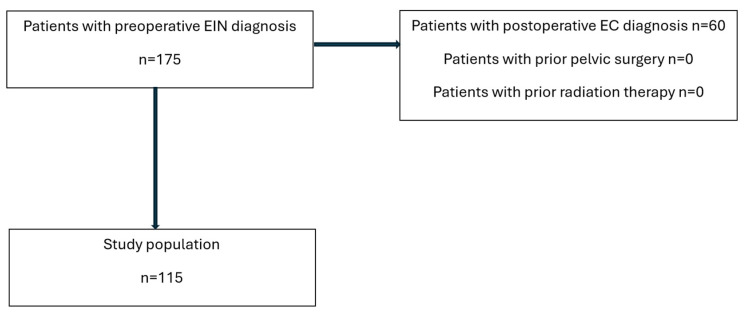
Flow diagram of patient selection and exclusion criteria.

This retrospective cohort study included 115 patients diagnosed with EIN at our institution between June 2009 and January 2023. EIN diagnosis was established based on endometrial biopsy, dilatation and curettage, or hysteroscopy, with final histopathologic examination confirming the absence of invasive carcinoma. Patients who underwent robotic hysterectomy were included. The exclusion criteria included patients with prior pelvic surgery or previous radiation therapy, and proven cancer in the final pathology (see Appendix A, Figure A1). When final histology revealed EIN, patients were redirected to their primary physician. This study received approval by the Institutional Review Board of the Jewish General Hospital, Montreal (protocol code #2020-1904, approval obtained 23 August 2019) with annual reviews (approval date until 20 August 2026) and complied with the Declaration of Helsinki. Consent was obtained from all patients prior to surgery.

### 2.1. SLN Procedure

The SLN procedure was performed according to our established protocol [11,17,18] and to Barlin and colleagues [19], based on our prior data showing minimal morbidity and short surgical time even with patients at a higher risk of complications [17,18]. Intra-cervical injections of indocyanine green (ICG) for near-infrared (NIR) fluorescence imaging were performed at the 3 and 9 o’clock positions, with both deep and superficial injections. SLNs were identified, excised, and subsequently examined by experienced gynecologic pathologists. In cases where SLNs were not successfully mapped, completion of LND was not performed.

### 2.2. Data Collection

Patient data was collected, including age, BMI (calculated as weight in kilograms divided by height in meters squared), menopausal status, surgical details (operative time, estimated blood loss), and final pathology reports (including details of any carcinoma found). All demographic, clinical, operative, and pathologic data has been prospectively recorded in a clinical database, which served as the data source for this study.

### 2.3. Statistical Analysis

Descriptive values were expressed as mean ± standard deviation (SD) or as median with a range. Descriptive statistics were utilized to summarize patient characteristics and SLN detection rates. BMI was categorized into subgroups according to the World Health Organization (WHO) classification. For categorical variables, chi-squared and Fisher tests were used to evaluate differences between groups (utilized specifically for comparison of SLN detection rates among the BMI categories). Distribution normality was assessed using the Kolmogorov–Smirnov test. Two-tailed Student’s *t*-tests were used to compare continuous variables normally distributed, and the Mann–Whitney U-test for comparing non-parametric continuous variables. Logistic regression analysis was employed to assess the independent association between BMI and SLN detection rate, adjusting for potential confounders such as age, tumor grade, and surgical experience. Statistical analysis was performed using the IBM SPSS Statistics software (version 27; IBM Corporation, New York, NY, USA). A *p*-value < 0.05 was considered statistically significant.

## 3. Results

**Figure 1:**
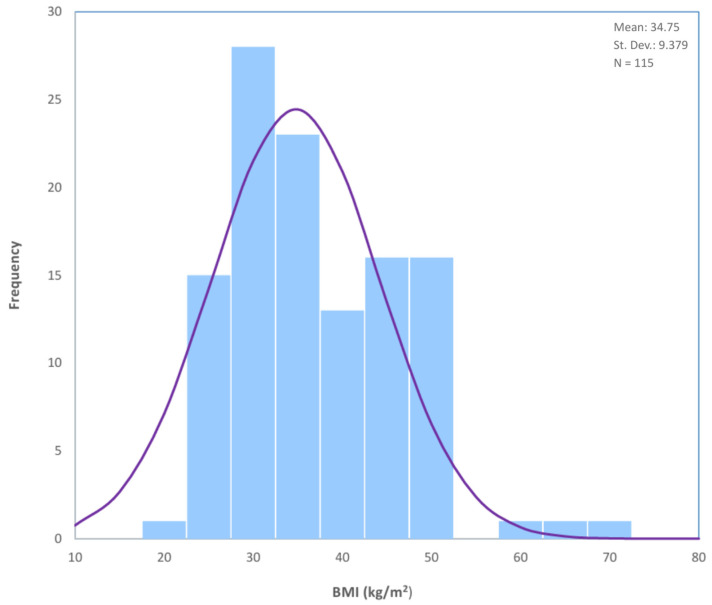
BMI Distribution in Study Population: Histogram depicting the distribution of BMI in the population (N = 115).

A total of 115 patients with the diagnosis of EIN were included in our study, with a mean BMI of 34.75 ± 9.38 SD (Table 1, Figure 1). The population characteristics are further detailed in Table 1.

### BMI Subgroup Analysis

SLN DRs were analyzed across the following BMI subgroups (World Health Organization classification): 24.9≥ (Normal Weight), 25–29.9 (Overweight), 30–34.9 (Obese Class I), 35–39.9 (Obese Class II), ≥40 (Obese Class III). The overall bilateral SLN DR was 73% (Table 2). The bilateral SLN DR for each BMI subgroup were as follows: Normal Weight: 87.5%, Overweight: 76%, Obese Class I: 68%, Obese Class II: 75%, Obese Class III: 67.6%% (Table 2). A chi-square analysis of all BMI subgroups revealed no significant difference in bilateral SLN DR among them (*p* = 0.606). Moreover, when specifically evaluating high BMI subgroups, no significant difference was observed either: a DR of 68.9% was found in patients with BMI > 30 (N = 74, *p* = 0.181), and a DR of 67.6% in those with BMI > 40 (N = 37, *p* = 0.362). Furthermore, a logistic regression analysis revealed that for every unit of BMI, the likelihood of bilateral SLN DR did not change significantly (adjusted odds ratio = 0.98, 95% CI 0.94–1.02, *p* = 0.321).

Using the same statistical methods for the analysis of unilateral SLN DR, the overall DR was 89.6%. The unilateral SLN DR for each BMI subgroup were as follows: Normal Weight: 100%, Overweight: 96%, Obese Class I: 84%, Obese Class II: 91.7%, Obese Class III: 83.8% (Table 3). A chi-square analysis comparing unilateral SLN detection rates across BMI subgroups showed no significant difference between BMI sub-groups (*p* = 0.269). Similarly, no significant difference was reported when examining high BMI sub-groups: a DR of 85.1% was found for BMI > 30 (N = 74, *p* = 0.053), and a DR of 83.8% for BMI > 40 (N = 37, *p* = 0.162). Again, a logistic regression was performed, showing that for every unit of BMI, the likelihood of unilateral SLN DR did not significantly change (adjusted odds ratio = 0.975, 95% CI 0.905–1.050, *p* = 0.5).

## 4. Discussion

The role of LN assessment and the performance of SLN in EIN cases are still controversial and, in the last few years, have been topics of debate amongst experts. Although the risk of LN involvement in EIN is low and little is known about the patterns of lymph node evaluation among patients with EIN, there are several compelling reasons to include LN assessment in the staging procedure. Firstly, assessing lymph nodes may be instrumental in guiding treatment decisions if EC is identified in the final pathology. This is especially important given that there is a risk of occult carcinoma at the time of EIN diagnosis, with studies indicating that up to 36% of cases may have a concurrent grade 2–3 tumor, while up to 47% may exhibit deep myometrial invasion (stages IB and IC) [7,8]. Additionally, in the case of EC reported in the final pathology, delayed LN assessment through an additional surgery may prove to be more challenging due to lymphatic disruption post-hysterectomy. Moreover, the fact that SLN biopsy has low morbidity and a high negative predictive value is an additional consideration supporting LN assessment in these cases [19]. On the other hand, given that LND does not necessarily improve disease-free or overall survival in EC [9], and given the decreased risk of LN involvement [4,8,20], other experts choose not to implement LND or SLN biopsy in EIN cases.

Since SLN biopsy became a useful tool in EC cases, several factors such as tumor histology, tumor grade, LN macro-metastasis, SLN sites, and history of prior pelvic surgery have been associated with reduced SLN DR and accuracy [12,13,14,15]. BMI and obesity, although widely believed to be factors which influence SLN DR, are still a topic of debate due to differing results in the literature. Body et al. used ICG for SLN mapping with a bilateral mapping success rate of 74% [21]. These authors noted that FIGO stage III or IV was significantly associated with decreased successful bilateral detection [21]. Age, BMI, previous C-section, grade, lymph-vascular space invasion, and cervical stromal invasion had no influence on this finding. In a meta-analysis exploring SLN assessment in EC, 55 studies were examined (including 4915 women) [22]. This analysis, which elucidated factors affecting SLN detection rate, found no correlation between high BMI and a decrease in SLN DR [22].

Conversely, one of the most comprehensive studies that explored the effect of BMI level on the SLN accuracy is the ObeLyX study. In this study, Vargiu et al. showed that the risk of SLN mapping failure has a 1.156-fold increase for every 5 units of BMI increase [23]. Though this study included laparoscopy and robotic surgery, the majority of patients with a BMI > 30 underwent robotic surgery. In their analysis, no subgroup analysis based on the surgical approach was performed for DR, although no correlation was found with the failure to find lymphatic tissue in the pathological sample [23]. Alternatively, Iavazzo et al. developed the “Iavazzo score”, a preoperative tool including BMI, uterine size, surgical history, vaginal parity and pathology, and it has been suggested that it could be used for the optimal preoperative patient selection [24].

Few studies were conducted on the effect of BMI specifically in robotic surgeries for EC. One of the first groups to address this topic was Eriksson et al. This group evaluated the DR in both ICG and blue dye, with bilateral DR of 75%, and unilateral DR of 15% [15]. Using ICG, a DR of 85% was achieved [15]. Patients with successful bilateral mapping had a median BMI of 29.8 kg/m^2^, compared with patients with unsuccessful mapping, in whom the median BMI was 34.7 kg/m^2^ [15]. There was a significant decrease in the rate of successful bilateral mapping for both the ICG (*p* < 0.001) and blue dye groups (*p* = 0.041) with increasing BMI [15]. One of the limitations mentioned in the study was the limited number of patients in BMI ≥40 group [15]. Another study conducted by Johnson et al. showed that patients with a BMI > 40 were found to have a significantly lower success rate for SLN mapping when compared with all other lower BMI categories (54.1% vs. 76.1%, respectively, *p* < 0.01) [25].

In order to examine the subject of SLN in EIN cases, Dioun et al. performed a comprehensive analysis of the “Premier Perspective Healthcare” database, which provides de-identified information on patient services in hospitals in the United States. In their work, they examined the utilization, morbidity, and cost of SLN mapping in 10,266 patients undergoing hysterectomy for EIN between January 2012 and June 2018 [26]. Their data suggests that SLN mapping for women with EIN has increased 17.5-fold in a 6 year span [26]. While they did not document the clinical utility of SLN mapping, the study suggested that this procedure has no association with increased perioperative mortality or increased morbidity [26]. Their cost-effectiveness analysis revealed that laparoscopic hysterectomy appeared to be less costly than either abdominal or robotic-assisted hysterectomy, but the addition of SLN mapping to laparoscopic hysterectomy increased its cost by 28% [26]. On the other hand, for robotic-assisted hysterectomies, the addition of SLN mapping had a minimal effect on the cost of surgery [26].

At our institution, the implementation of SLN assessment in robotic surgery has become standard of care for patients with EIN since 2010. Matanes et al., in a previous publication from our institution, found that 61 out of 162 patients (37.7%) initially diagnosed with EIN were ultimately found to have EC on final pathology [16]. Of those, 15 patients (9.25% of the initial EIN diagnoses) had high-intermediate to high-risk EC [16]. Of the 61 patients found to have EC, ten patients had negative SLN, which allowed those patients to receive only adjuvant vaginal brachytherapy [16]. Without the information provided by the SLN, these patients (who possessed multiple high-risk factors, including grade 2 cancer with >50% myometrial invasion) would have likely been candidates for more extensive and potentially more morbid treatment regimens including external beam pelvic radiotherapy [16]. Five of the 61 EC patients with positive SLNs (micrometastases), required a more extensive therapeutic approach that may have been delayed or avoided if the SLN assessment had not been performed [16]. In these situations, the SLN provided crucial information for appropriately tailoring the therapeutic intervention. Without SLN information, treatment decisions might have been suboptimal.

In light of the adoption of SLN assessment in EIN cases and the discussion around this topic, our study aimed to investigate the association between BMI and SLN detection rate in patients undergoing robotic hysterectomy for EIN. This relationship is particularly relevant given the well-established link between high BMI and the risk for EC and EIN [27,28,29,30,31]. Indeed, mechanisms such as hyperinsulinemia, inflammation, production of endogenous estrogens within the adipose tissue, and altered growth factor signaling all drive the increased risk of endometrial cancer in obese individuals [32,33]. This risk was found to be overt not only in postmenopausal patients, but also in premenopausal patients [34].

Contrary to some reports in the literature suggesting a negative correlation between high BMI and SLN detection in endometrial cancer, our findings demonstrate no statistically significant association between BMI and SLN detection rate across a broad range of BMI categories, including obesity and morbid obesity. This result holds true even when considering unilateral SLN detection rates. We also failed to find a specific BMI value that can be used as a “cut-off” value from which any addition of BMI unit can cause a change in the SLN detection rate. These findings are in line with some studies investigating this issue in EC cases. The absence of a significant association, even after adjusting for potential confounders such as age, parity, gravity and relevant past medical history (e.g., diabetes and hypertension), strengthens the robustness of our findings. One strength of this study is the fact that all surgeries were performed by the same 3 surgeons, providing maximal consistency with reduced variability and bias. The overt limitations of our study include its retrospective design and relatively small sample size, which may introduce potential biases including, but not limited to, patient selection bias, and institutional policies and procedures that could influence SLN DR.

Our results have important clinical implications, suggesting that BMI does not appear to significantly influence the efficacy of SLN biopsy in robotic surgery for EIN. Therefore, SLN biopsy may be a reliable staging technique in these patients regardless of BMI. To the best of our knowledge, no similar study on the influence of BMI on the detection rate of SLN during robotic surgery in the treatment of EIN has been published. For our results to be generalizable, future research should focus on larger prospective studies to validate our findings. Indeed, a multi-center study could more appropriately account for differences in surgeon skill, robotic platform utilization and differences in institutional practices, thereby allowing the results to be transferable to smaller centers with lower surgical volumes. While multiple studies suggest that SLN evaluation in EIN cases would offer beneficial guidance to clinical decision-making [16,26], its significance in predicting recurrence rates, upstaging of disease or survival has yet to be characterized. Studies investigating the potential for predictive models combining BMI with other clinical parameters to improve SLN detection rate prediction could also provide valuable clinical guidance.

## 5. Conclusions

Obesity (BMI > 30) and morbid obesity (BMI > 40) do not appear to be associated with a reduced SLN DR in EIN cases. There also appears to be no significant variation in SLN DR across all the BMI categories. These results support the feasibility of SLN assessment during robotic hysterectomy in patients with EIN regardless of their BMI status and may inform further treatment and decision-making.

## Figures and Tables

**Table 1:** Population Baseline Characteristics.

Parameter	Value	Mean	Median	Min	Max	Range	SD
Total Number of Patients (n)	115						
Age (years)	-	61	61	40	86	46	±10
BMI (kg/m^2^)	-	34.75	33.0	18.9	67.0	48.1	±9.38
BMI Categories (kg/m^2^)							
Normal (24.9≥)	16 (13.9%)						
Overweight (25.0–29.9)	25 (21.7%)						
Obese Class I (30.0–34.9)	25 (21.7%)						
Obese Class II (35.0–39.9)	12 (10.4%)						
Obese Class III (≥40)	37 (32.2%)						
Diabetes Mellitus	22 (19.3%)						
Hypertension	54 (47%)						
ASA Score							
ASA I	20 (17.4%)						
ASA II	53 (46.1%)						
ASA III	41 (35.7%)						
unavailable	1 (0.9%)						
Gravidity							
0	17 (14.8%)						
1	15 (13%)						
2	44 (38.3%)						
3	25 (21.7%)						
>3	14 (12.2%)						
Parity							
0	22 (19.1%)						
1	16 (13.9%)						
2	50 (43.5%)						
3	25 (21.7%)						
>3	2 (1.8%)						

**Table 2:** Comparison of bilateral SLN detection rate across five different BMI subgroups.

BMI Group	Not Detected	Detected	Total	Non-Detection Rate (%)	Detection Rate (%)
Normal (24.9≥)	2	14	16	12.5	87.5
Overweight (25.0–29.9)	6	19	25	24	76
Obese Class I (30.0–34.9)	8	17	25	32	68
Obese Class II (35.0–39.9)	3	9	12	25	75
Obese Class III (≥40)	12	25	37	32.4	67.6
Total	31	84	115	27	73

**Table 3:** Comparison of unilateral SLN detection rate across five different BMI subgroups.

BMI Group	Not Detected	Detected	Total	Non-Detection Rate (%)	Detection Rate (%)
Normal (24.9≥)	0	16	16	0	100
Overweight (25.0–29.9)	1	24	25	4	96
Obese Class I (30.0–34.9)	4	21	25	16	84
Obese Class II (35.0–39.9)	1	11	12	8.3	91.7
Obese Class III (≥40)	6	31	37	16.2	83.8
Total	12	103	115	10.4	89.6

## Data Availability

The raw data supporting the conclusions of this article will be made available by the authors on request.

## Abbreviations

The following abbreviations are used in this manuscript:

BMI	Body-mass index
DR	Detection rate
EC	Endometrial carcinoma
EIN	Endometrial intraepithelial neoplasia
ICG	Indocyanine green
LN	Lymph node
LND	Lymph node dissection
NIR	Near-infrared
SD	Standard deviation
SLN	Sentinel lymph node
